# Workers’ Health Surveillance in the Meat Processing Industry: Work and Health Indicators Associated with Work Ability

**DOI:** 10.1007/s10926-015-9569-2

**Published:** 2015-02-13

**Authors:** Berry J. van Holland, Remko Soer, Michiel R. de Boer, Michiel F. Reneman, Sandra Brouwer

**Affiliations:** 1 Department of Rehabilitation Medicine, Center for Rehabilitation, University Medical Center Groningen, University of Groningen, Dilgtweg 5, 9751 ND Haren, The Netherlands; 2 Expertise Center of Health, Social Care and Technology, Saxion University of Applied Sciences, Enschede, The Netherlands; 3 Groningen Spine Center, University Medical Center Groningen, University of Groningen, Groningen, The Netherlands; 4 Department of Health Sciences, Faculty of Earth and Life Sciences, Institute for Health Sciences, VU University, Amsterdam, The Netherlands; 5 Department of Health Sciences, Community and Occupational Medicine, University Medical Center Groningen, University of Groningen, Groningen, The Netherlands

**Keywords:** Occupational health, Health risk appraisal, Work ability, Functional capacity evaluation, Meat-packing industry

## Abstract

*Background* Workers’ health surveillance (WHS) programs commonly measure a large number of indicators addressing health habits and health risks. Recently, work ability and functional capacity have been included as important risk measures in WHS. In order to address work ability appropriately, knowledge of associations with work and health measures is necessary. The objective of this study was to evaluate which of the factors measured in a WHS are independently associated with work ability in a group of meat processing workers. *Methods* A cross-sectional study was performed in a large meat processing company in The Netherlands. Data were collected during a WHS between February 2012 and March 2014. Personal characteristics, health habits and health-risk indicators, functional capacity, and work-related factors were measured. Work ability was measured with the Work Ability Index and was used as dependent variable. Univariable and multivariable logistic regression analyses were conducted, a receiver operating characteristic curve was constructed and the area under the curve (AUC) was calculated. *Results* Data sets from 230 employees were used for analyses. The average age was 53 years and the average work ability index score was 39.3. In the final multivariable model age (OR 0.94), systolic blood pressure (OR 1.03), need for recovery (OR 0.56), and overhead work capacity (OR 3.95) contributed significantly. The AUC for this model was 0.81 (95 % CI 0.75–0.86). *Conclusion* Findings from the current study indicate that multifactorial outcomes (age, systolic blood pressure, need for recovery, and overhead work capacity) from a WHS were independently associated with work ability. These factors can be used to assess employees at risk for low work ability and might provide directions for interventions.

**Electronic supplementary material:**

The online version of this article (doi:10.1007/s10926-015-9569-2) contains supplementary material, which is available to authorized users.

## Introduction

Over the past decades, the number of organizations and companies that offer workers’ health surveillance (WHS) for their employees at the worksite has increased [1, 2]. The overall aims of a WHS as part of health promotion programs are prevention of occupational illnesses and work-related injuries, maintenance and promotion of health in relation to work, and maintenance and improvement of functioning and employability [3, 4]. Through early detection and intervention for health issues a WHS aims to prevent the development of an occupational or work-related disease that may affect the workers’ functioning [5]. WHSs have been conducted in many different ways and with different aims, however, the basic elements of WHSs are “the assessment of personal health habits and health risk factors; a quantitative estimation or qualitative assessment of future risk of death and other adverse health outcomes; and provision of feedback in the form of educational messages and counseling that describe ways in which changing one or more behavioral risk factors might alter the risk of disease or death” [6–8]. More recently, WHSs also include assessments of work ability to detect and identify any signals of change in health and functioning. Work ability levels have been found to be an important indicator of the balance between human resources and work demands [9, 10], and have been shown to be strongly associated with risk of sickness absence and work disability [11–13]. Although several studies have investigated factors influencing work ability, only few studies have estimated to what extent different WHS indicators contribute to the level of work ability.

WHSs are often applied in certain occupations where workers are known to be at risk for adverse health effects due to their occupational exposure (p 11 in [5]). Previous studies have described job-specific WHSs for painters [14], fire fighters [15], agriculture workers [16], nurses [17] and construction workers [5]. Another field in which workers are known to be at high risk for adverse health effects due to occupational exposure is the meat processing industry. Meat processing workers perform monotonous and physically demanding work [18, 19] and are a vulnerable group in the way that they are exposed to several occupational health hazards simultaneously. Most common occupational injuries and illnesses reported are musculoskeletal disorders (MSDs), skin disorders, hearing disorders and infectious diseases. Prevalence rates of MSDs of over 90 % have been reported [20, 21]. In general, it is known that these diseases increase the chance for sickness absence and reduced work ability [22]. If injuries or illnesses are severe enough, they may lead to early retirement or disability benefit [23] and thus have considerable economic consequences [24].

In 2011 a large Dutch meat company introduced the POSE program (Promotion Of Sustained Employability) [25], a WHS combining elements from occupational (e.g. health surveillance, and interventions aimed at a healthy lifestyle) and rehabilitation medicine [e.g. Functional Capacity Evaluation (FCE) tests, and interventions aimed at improved physical capacity]. The POSE program aims to offer employees a custom-made risk profile and, if necessary, an intervention plan using an integral approach [26].

Although in most research on WHS and workplace health promotion programs the focus is on investigating its effectiveness on health and work functioning outcome measures, more knowledge should also be obtained about the relevance of the gathered information by the included tests and assessments. This knowledge is essential for the selection of appropriate surveillance instruments [27], meaning that only relevant aspects should be addressed and workers should be protected from an abundance of screening tests. The better the surveillance is attuned to the requirements of the occupation and the needs of the workers, the better the possibility to develop and deploy effective interventions [5, 28]. The aim of the present study was to examine which indicators from a health-risk assessment and functional capacity evaluation tests are independently associated with work ability in a sample of meat processing workers.

## Methods

The STROBE statement was followed to report this study [29], which is a guideline for the reporting of observational studies, including things like data sources, statistical methods, main results, and generalizability.

### Study Design and Setting

This study was designed as a cross-sectional study which was carried out at three plants of a large meat processing company in The Netherlands. Data were collected during four WHSs between February 2012 and March 2014. The study was part of the FLESH study (Functional Labor Evaluation for Sustained Health and employment) which has been described elsewhere [25].

### Participants

Four groups of participants were recruited. They were eligible if they were contracted production personnel and worked at least 12 h per week, participated in a WHS, and provided informed consent to participate in the study.

### Measurements

#### Dependent Variable

Work ability was the outcome of interest and was assessed in the WHS. It was measured by the Work Ability Index (WAI) [30] which consists of 28 questions focusing on mental and physical work ability, injuries and diseases, and future work expectations. Sum score can range from 7 to 49, with higher scores indicating better work ability. Scores were divided into four categories: 7–27 (poor), 28–36 (moderate), 37–43 (good), and 44–49 (excellent) [31].

#### Independent Variables

Work-related factors were assessed in the WHS. Three constructs from the Dutch Questionnaire on the Experience and Evaluation of Work (VBBA) [32] were measured during the WHS. One question asked whether employees considered their work physically demanding (no/yes). Another question asked whether employees considered their work mentally too demanding (no/yes). Five items evaluated the Need For Recovery after work (NFR) (no/yes) [33], resulting in a scale score ranging from 0 to 5. Higher scores indicated more need for recovery after work. Cronbach’s alpha for the NFR scale was 0.72. The number of contractual work hours per 4 weeks was retrieved from company administration.

A Functional Capacity Evaluation was performed. Eight domains of functional capacity (lifting low, lifting high, carrying, repetitive bending, repetitive side reach, overhead work, forward bent work, and hand strength) were tested, largely based on the WorkWell FCE [34]. Exact procedures and normative values have been described elsewhere [35]. Results were categorized according to cut-off values for each domain [36, 37]. Workers scored sufficient capacity if their score was higher than this cut-off value (Appendix 1 in ESM). A submaximal cycling test was used to estimate maximal aerobic capacity ($\mathop {\text{V}}\limits^{.} {\text{O}}_{2} \hbox{max}$) [38]. Participants cycled for 6–7 min on an electromagnetically braked cycle ergometer (Tunturi E80, Tunturi, Bergeijk, The Netherlands) with a target heart rate above 120 beats per minute. Based on power output, age, body weight, and heart rate, the $\mathop {\text{V}}\limits^{.} {\text{O}}_{2} \hbox{max}$ was estimated. A cut-off value of 32.9 ml O_2_/min/kg was applied to categorize the outcome.

Health-risk factors were assessed in the WHS. Body length in cm and weight in kg (Seca 217 and Seca 803, Seca Deutschland, Hamburg, Germany) were measured. Body fat percentage was measured with a handheld electronic device (Omron BF306, Cemex B.V., Nieuwegein, The Netherlands). Systolic and diastolic blood pressure (BP) and resting heart rate were measured by an automatic sphygmomanometer (Omron PA-350-E, Cemex B.V., Nieuwegein, The Netherlands). Blood glucose level (mmol/l) and total cholesterol (mmol/l) were measured from a small blood sample from the fingertip (Accutrend Plus, Roche Diagnostics Nederland B.V., Almere, The Netherlands). Smoking was assessed by the question “Do you smoke at this moment” (no/yes). Alcohol use was assessed by the question “Do you occasionally use alcohol” (no/yes). Eating habits were assessed by the question “Do you consider yourself having healthy eating habits” (no/yes).

Personal characteristics (age in years, gender, and affiliation duration in years) were retrieved from company administration. Educational level was assessed in the WHS and was categorized as low (no education/elementary school/preparatory secondary vocational education), or medium–high (secondary vocational education/higher secondary education/higher professional education/university).

### Statistical Analysis

Production personnel with data from the WHS, company administration, and questionnaire were included in the analyses. Not all participants provided complete data. We therefore decided to impute data using chained imputations [39] with an imputation model consisting of all the potential predictors, the dependent variables and three other variables which we thought were related to missingness. These three variables were work location, number of absence days and absence episodes in the year before the WHS. Trace plots of means and standard deviations of imputed variables were checked for convergence. It was found that results were stable after 50 imputations, which were used in the final analyses. Based on pooling of the estimates of these 50 imputed databases, first descriptive analyses were performed. Next, univariable as well as multivariable ordinal regression models with the WAI as the dependent and the health-risk assessment variables (work-related factors, functional capacity, health-risk factors, and personal characteristics) as independent variables were constructed. In the multivariable analyses a forward method was used for the selection of variables in the final model. All indicators were stepwise entered in the multivariable model and included if *p* < 0.05. Associations were considered significant at *p* < 0.05. To assess the discriminative value of the multivariable model, a receiver operating characteristic (ROC) curve was constructed and the area under the curve (AUC) was calculated. All analyses were carried out using STATA version 12.1 (StataCorp, College Station, Texas, USA).

#### Sensitivity Analysis

From observations at the different company plants it was found that the participating employees hardly performed work near or over cut-off values of some FC tests (lifting low, lifting high, carrying). Since we had data available on the true workload, obtained during workplace observations, we decided to perform a sensitivity analysis with lower cut-off values for these tests. These lower values reflect the upper limits of real-day workload. For lifting low and carrying the cut-off value was set at 22.7 kg and for lifting high at 15.0 kg. Again, univariable and multivariable analyses were performed.

## Results

A total of 245 production workers participated in the WHS. Eighty-two employees came from plant A, 126 (divided in groups of 70 and 56) from plant B, and 37 from plant C. In the process of imputing data, we had to remove 15 subjects from the dataset to enable convergence. These fifteen persons had missing values on all of the VBBA items. After imputation of missing data, complete datasets were available from 230 employees, which were used for the analyses in this study.

A descriptive overview of the sample characteristics is provided in Table 1. An overview of the raw (non-imputed) data is presented in Appendix 2 in ESM. The majority of the subjects (90 %) are men and the average age for the total sample is 53 years. On average the employees had worked at the company for 22.5 years. The average score on the Work Ability Index was 39.3. The distribution of work ability across categories is displayed in Table 1. Based on the distribution across categories (skewed to the right), we decided to combine the two lowest categories into a poor-moderate group (n = 63; 27 %) and the two highest categories into a good–excellent group (n = 167; 73 %). Instead of ordinal regression analyses, logistic regression analyses were conducted.

**Table 1:** Overview of outcomes for the total sample (N = 230) and for both WAI categories

	Total		WAI+		WAI−	
	Mean/*N*	SD/ %	Mean/*N*	SD/ %	Mean/*N*	SD/ %
Work Ability Index (7–49)	39.3	5.4	41.9	3.2	32.3	3.7
	230	100 %	167	73 %	63	27 %
Poor (7–27)	7	3.0 %				
Moderate (28–36)	56	24.3 %				
Good (37–43)	115	50.0 %				
Excellent (44–49)	49	21.3 %				
Missing	3	1.3 %				
Personal characteristics						
Gender (% male)	206	90 %	149	89 %	57	90 %
Age (year)	52.9	6.7	52.2	6.7	54.5	6.4
Affiliation duration (year)	22.5	10.7	22.0	10.7	23.9	10.6
Contract hours/4 weeks (h)	141.6	15.1	141.7	15.1	141.6	15.3
Educational level, low	171	74 %	123	74 %	48	76 %
Biometric data						
Cholesterol (mmol/l)	5.3	0.9	5.3	0.9	5.4	0.9
Glucose (mmol/l)	5.9	1.8	5.8	1.8	6.1	1.6
Systolic BP (mm Hg)	140.7	18.5	142.0	19.2	137.3	15.8
Diastolic BP (mm Hg)	82.7	10.1	82.6	10.5	82.7	9.0
Resting heart rate (bpm)	71.4	12.3	71.5	12.1	71.3	13.2
Body length (m)	175.6	8.8	176.0	8.7	174.7	9.1
Body weight (kg)	85.9	16.0	85.3	16.3	87.5	15.6
Fat percentage (%)	27.4	7.2	26.9	7.4	28.6	6.6
Health						
Smoking, yes	95	41 %	70	42 %	25	40 %
Alcohol use, yes	178	77 %	133	80 %	45	72 %
Healthy eating habits, yes	110	48 %	79	47 %	31	49 %
Functional capacity^a^						
Aerobic capacity (ml/min/kg)	30.8	9.8	31.3	10.1	29.4	8.7
>32.9 ml/min/kg	90	39 %	70	42 %	20	31 %
Lifting low (kg)	32.6	12.1	34.1	11.9	28.7	11.2
>45 kg	17	7 %	–	–	–	–
Lifting high (kg)	17.0	6.5	17.4	6.4	15.8	6.1
>24 kg	18	8 %	14	8 %	4	6 %
Carrying (kg)	37.1	11.5	38.0	11.6	34.8	10.6
>48 kg	20	8 %	16	10 %	4	6 %
Overhead work (s)	220.9	98.2	237.6	92.7	177.0	96.4
>221 s	118	51 %	102	61 %	16	26 %
Forward bent work (s)	244.5	100.5	255.1	95.1	216.7	103.1
>262 s	121	53 %	98	58 %	23	37 %
Repetitive bending (s)	47.9	10.3	47.1	9.7	49.8	11.2
<55 s	191	83 %	141	84 %	50	80 %
Trunk rotation right (s)	68.5	15.2	67.2	14.6	71.8	15.5
<93 s	219	95 %	161	96 %	58	92 %
Trunk rotation left (s)	68.5	13.7	67.3	13.1	71.7	14.1
<98 s	222	97 %	164	98 %	58	92 %
Hand grip strength (kgf)	49.2	11.1	49.5	11.0	48.3	11.3
>32.5 kg	213	92 %	156	93 %	57	90 %
VBBA						
High physical workload, yes	100	43 %	66	40 %	34	54 %
High mental workload, yes	40	17 %	25	15 %	15	24 %
Need for recovery (0–5)	1.2	1.4	0.9	1.2	2.1	1.7

Number of participants, number of participants with sufficient functional capacity and means (SD) are presented

WAI +, employees scoring equal to or above WAI cut-off; WAI−, employees scoring below WAI cut-off

*WAI* Work Ability Index, *VBBA* Dutch Questionnaire on the Experience and Evaluation of Work

^a^For functional capacity the number of participants scoring better than the cut-off value is presented together with average (SD) scores on these tests

The results from the univariable analyses are displayed in Table 2. Significant associations with the WAI were observed for 5 variables: age (Odds ratio (OR) 0.95), need for recovery (OR 0.57), overhead work (OR 4.36), forward bent work (OR 2.38), and trunk rotation left (OR 5.50). OR’s indicate that lower age, lower need for recovery, sufficient overhead work capacity, sufficient forward bent work capacity, and sufficient left trunk rotation capacity are related to a good WAI score. No odds ratio could be calculated for ‘lifting low’, because pooling of effect estimates for the imputed datasets was not possible. Besides these 5 variables, no other personal characteristics, biometric characteristics, health habits, functional capacity, or work-related characteristics were significantly associated with the WAI.

**Table 2:** Odds ratios (ORs), their 95 % confidence interval (95 % CI), and *p* values for having good to excellent work ability: results from the univariable analyses

	OR	95 % CI		*p*
		LL	UL	
Personal characteristics				
Gender	1.16	0.44	3.06	0.770
Age	0.95	0.90	0.99	0.024
Affiliation duration	0.98	0.96	1.01	0.227
Contract hours	1.00	0.98	1.02	0.980
Educational level	1.14	0.58	2.24	0.705
Biometric data				
Cholesterol	0.97	0.69	1.35	0.834
Glucose	0.91	0.76	1.07	0.256
Systolic BP	1.02	1.00	1.03	0.080
Diastolic BP	1.00	0.97	1.03	0.937
Resting heart rate	1.00	0.98	1.03	0.924
Body length	1.02	0.98	1.05	0.320
Body weight	0.99	0.97	1.01	0.359
Fat percentage	0.97	0.93	1.01	0.122
Health				
Smoking	1.06	0.56	2.03	0.850
Alcohol use	1.55	0.80	3.01	0.196
Healthy eating habits	0.92	0.52	1.65	0.782
Functional capacity				
Aerobic capacity	1.61	0.84	3.11	0.155
Lifting low	–	–	–	–
Lifting high	1.35	0.35	5.18	0.662
Carrying	1.68	0.39	7.22	0.482
Overhead work	4.36	2.14	8.88	0.000
Forward bent work	2.38	1.23	4.57	0.010
Repetitive bending	1.33	0.58	3.07	0.496
Trunk rotation right	2.27	0.62	8.22	0.214
Trunk rotation left	5.50	1.05	28.76	0.043
Hand grip strength	1.49	0.52	4.21	0.457
VBBA				
High physical workload	0.56	0.31	1.00	0.052
High mental workload	0.55	0.27	1.14	0.109
Need for recovery	0.57	0.46	0.71	0.000

*LL* Lower limit, *UL* Upper limit, *VBBA* Dutch Questionnaire on the Experience and Evaluation of Work

**Fig. 1:**
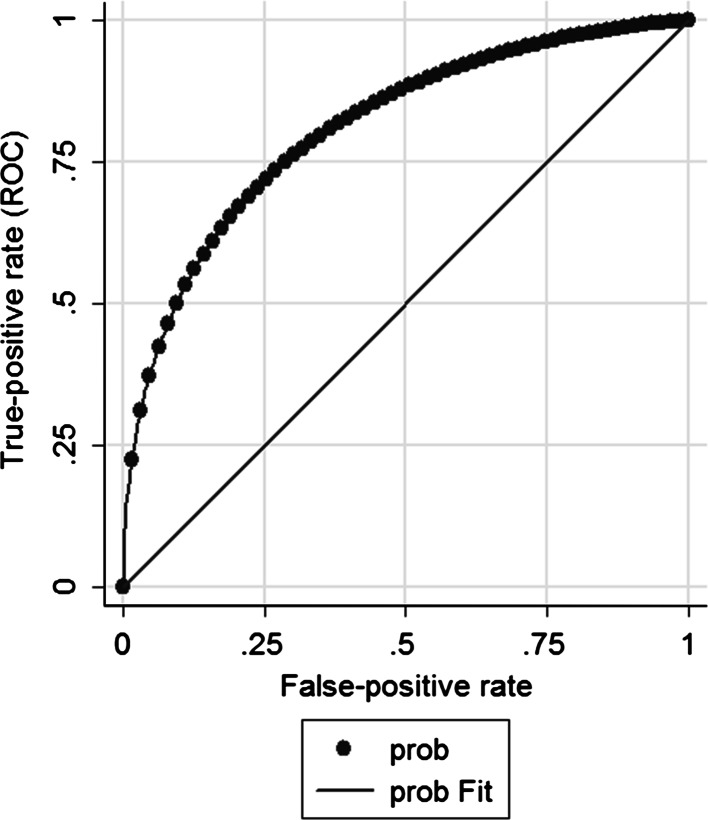
ROC curve for the multivariable model predicting good to excellent work ability. X-axis: probability of false-positive predictions; Y-axis: probability of true-positive predictions. AUC = 0.81 (95 % CI 0.75–0.86)

When all variables were stepwise entered into a multivariable logistic regression model, 4 variables independently contributed significantly: age, systolic BP, need for recovery, and overhead work (Table 3). OR’s indicate that lower age, higher systolic blood pressure, lower need for recovery, and sufficient overhead work capacity lead to a good WAI score. The AUC for this model was 0.81 (95 % CI 0.75–0.86) (Fig. 1). The formula for work ability level (poor/good), which can be derived from the ORs, is as follows: 0.968 − 0.579 * NFR + 1.374 * overhead work − 0.066 * age + 0.027 * systolic BP.

**Table 3:** Odds ratios (ORs), their 95 % confidence interval (95 % CI), and *p* values for having good to excellent work ability: results from the multivariable analyses

Multivariable model	OR	95 % CI		*p*
		LL	UL	
Need for recovery	0.56	0.44	0.71	0.000
Overhead work	3.95	1.80	8.68	0.001
Age	0.94	0.89	0.99	0.016
Systolic BP	1.03	1.00	1.05	0.025

Overhead work is included as dichotomous variable; need for recovery, age, and systolic BP are included as continuous variables

*LL* lower limit, *UL* upper limit

### Sensitivity Analysis

For three FC tests (lifting low, lifting high, and carrying) additional univariable analyses with lower cut-off values were performed. This resulted in changed ORs for lifting high (OR 1.61) and carrying (OR 1.41), but they were still not significant. For lifting low pooling of effect estimates was now possible, which resulted in an OR of 2.14 (95 % CI 1.01–4.52; *p* = 0.047) in the univariable analyses. None of these three variables contributed significantly to the multivariable model.

## Discussion

The results of this study showed that age, systolic blood pressure, need for recovery and overhead work were independently associated with work ability in a sample of meat processing workers. Being older decreased the odds for good work ability by 6 % per year. Higher systolic blood pressure, despite being significant, led to only 3 % higher odds for good work ability. In numbers, a 10 mmHg higher systolic blood pressure increased the odds for good work ability by 30 %. Workers with lower need for recovery had about twice the odds for good work ability as did workers with higher need for recovery. Workers with sufficient overhead work capacity had a four times higher odds for good work ability. Single indicators that showed significant associations with work ability were age, need for recovery, overhead work, forward bent work, and trunk rotation left.

Age was found to be significantly associated with work ability, where higher age is an indicator for lower work ability. This confirms findings from previous studies [40–42]. On the other hand, inconsistent relations between work ability and age have been reported [43]. From a scientific point of view it is interesting to incorporate factors such as age and gender in a model to explain work ability. From a practical point of view this may be debated, because both factors are not modifiable and therefore cannot be used as a basis for intervention. However, certain problems, e.g. musculoskeletal or cardiovascular, are age-related [44] and can be addressed in interventions for specific age-groups which in turn may positively influence work ability. A surprising finding was the significant association between systolic BP and work ability, especially the direction of the association, since high BP is normally considered as a health-risk indicator. An explanation might be that employees with poor work ability used medication against high BP, resulting in a lower systolic BP. As a consequence, lower work ability is associated with lower BP. Since the OR is very close to 1, and we identified no literature that higher SBP may be protective to work ability, the relation between SBP and work ability may be based on coincidence and has little or no clinical relevance. Unfortunately, our data did not contain enough information on medication use to check this assumption. Need for recovery after work was also found to be significantly associated with work ability. Although no previous studies were identified that investigated this association, similar results were reported for the relation between fatigue and work ability [45, 46]. Lack of recovery can lead to the onset of occupational injuries and illnesses [47, 48] and reduced work ability. Furthermore, as in previous studies, better functional capacity was positively associated with work ability [49–52], although in our model only one FCE test (overhead work) was included in the final model. This association is plausible since work in the meat processing industry is predominantly physical and this test resembles typical job tasks. Some other FCE tests were significantly associated with the WAI in the univariable analyses, but were not included in the multivariable model. This might be due to correlations with the variables included in the final model, e.g., forward bent work (r = −0.204) and trunk rotation left (r = −0.149) correlated significantly with NFR.

The ROC curve indicates that our model has good discriminating ability in terms of classifying workers into their respective work ability category. The best combination of sensitivity and specificity leads to a value of 0.72 for both, indicating that 28 % could be falsely categorized as having good–excellent or poor–moderate work ability. The model cannot completely explain worker classification, which implies that other indicators could be involved. Further study is necessary to identify these indicators. Furthermore, individual indicators and the WAI may contain measurement error in themselves. So, a certain amount of uncertainty in the model is inevitable.

## Strengths and Limitations

To our knowledge, this is the first study that investigates associations of various indicators with work ability in a WHS. The cross-sectional design of this study makes it impossible to draw conclusions on possible causal relationships. We therefore stress the need of longitudinal studies to elaborate on our findings. Other relevant outcomes could be included in those studies, such as sickness absence and productivity. Another point of interest is the homogeneity of the population. It consists of only blue collar workers from one meat processing company, which might be considered as a strength of this study. At the same time, homogeneity might be a drawback since it may hinder the explanation of variance and is a limitation concerning generalizability. Nevertheless, it is assumed that findings from this study may apply to production personnel outside the industry, but with similar job tasks.

A limitation of this study is the fact that employees could refuse to participate in the WHS. This might have caused a selection bias, since participants generally appear to be somewhat healthier than non-participants [53]. This may have resulted in the good average work ability of our study sample, and the above cut-off work ability of almost two-thirds of the sample. Nevertheless, the on average good work ability might be an overestimation of the true score, since workers with high physical work demands are less inclined to report a low work performance, compared to workers with more mentally demanding work [54].

For all FCE tests, participants were instructed to put in their maximum effort. However, it is possible that they did not reach their maximum [55], and as a consequence scored below cut-off. It is also possible that cut-off values were not applicable for this population, because the job demands were lower in general. The sensitivity analysis demonstrated that lower cut-off values for some tests did not change the multivariable model. It is possible for a trained observer to estimate whether maximum effort was put in [55], but this was not recorded for the current study.

Imputation of missing data was performed to get complete data for as many workers as possible. In the end, 245 production workers provided data on most or all variables. However, 15 workers lacked data on all VBBA items. These employees had to be excluded from the dataset, because analyses on imputed data did not lead to convergence. Descriptive analyses on both raw and imputed data from the remaining 230 participants show similar results. This implies that imputations were done reliably and valid conclusions can be drawn for the entire sample.

## Implications for Practice

The growing proportion of older workers in the meat processing industry stresses the need for new policies and programs to assure health and sustainable employability of the workers. In 2009 and 2010, the Dutch Labor Inspectorate performed nationwide inquiries at multiple meat processing companies. The main risk factors identified for sustainable employability were related to job demands and job design (machine handling, knife handling, repetitive movements, static postures, work pressure), and contextual factors (work on platforms, biologic agents, noise, safety measures) [56, 57]. The finding that age is related to work ability stresses the need for interventions aimed at sustainable employability specifically targeted on the aging population (44). This study also shows that to improve work ability, elements from occupational medicine as well as from rehabilitation medicine should be addressed. Furthermore, need for recovery after work should be addressed by, for example, the introduction of more rest-breaks during work [58, 59].

## Conclusion

In a WHS for meat processing workers, one socio-demographic indicator (age), one health-risk indicator (systolic blood pressure), one work-related indicator (need for recovery), and one functional capacity indicator (overhead work) were shown to be related to work ability. To confirm and expand our findings, longitudinal studies should be performed, incorporating other (health) outcomes as well.

## Electronic supplementary material

Below is the link to the electronic supplementary material.

Supplementary material 1 (DOCX 21 kb)
