# Supplementary material for: Workers’ Health Surveillance in the Meat Processing Industry: Work and Health Indicators Associated with Work Ability
Source: J Occup Rehabil. 2015 Feb 13;25(3):618–26. doi: 10.1007/s10926-015-9569-2 (PMC4540765; doi:10.1007/s10926-015-9569-2)
Supplement: Supplementary file 1 — Supplementary material 1 (DOCX 21 kb) [file 10926_2015_9569_MOESM1_ESM.docx]

**APPENDIX 1**

Cut-off values for Functional Capacity Evaluation.

**Scores expressed in kilograms**

| **FORCE** | **Criterion** |
| --- | --- |
| Lifting low | > 45 |
| Lifting high | > 24 |
| Carrying | > 48 |
| Hand grip strength | > 32.5 |

**Scores expressed in seconds**

| **STATIC WORK** | **Criterion** |
| --- | --- |
| Forward bent work | > 262 |
| Overhead work | > 221 |

**Scores expressed in seconds**

| **REPETITIVE WORK** | **Criterion** |
| --- | --- |
| Repetitive side reach |  |
| *Left* | < 98 |
| *Right* | < 93 |
| Repetitive bending | < 55 |

**Scores expressed in METs^*^ and VO_2_**

| **AEROBIC CAPACITY** | **Criterion** |
| --- | --- |
| Åstrand test (MET)* | > 9.4 |
| (VO_2_: ml/min/kg) | > 32.9 |

^* 1 MET = 3.5 ml O2/min/kg^

**Based on:**

Soer R, van der Schans CP, Geertzen JH, Groothoff JW, Brouwer S, Dijkstra PU, Reneman MF. Normative values for a functional capacity evaluation. Arch Phys Med Rehabil. 2009 Oct;90(10):1785-94. <http://dx.doi.org/10.1016/j.apmr.2009.05.008>

Hollak N, Soer R, Van der Woude LH, Reneman MF. Towards a comprehensive Functional Capacity Evaluation for hand function. Appl Ergon. 2014 May;(45(3):686-92. <http://dx.doi.org/10.1016/j.apergo.2013.09.006>

Soer R, Hollak N, Deijs M, Van der Woude LH, Reneman MF. Matching physical work demands with functional capacity in healthy workers: Can it be more efficient? Appl Ergon. 2014 Jul;45(4):1116-22. <http://dx.doi.org/10.1016/j.apergo.2014.01.011>

**APPENDIX 2**

Sample characteristics from raw (non-imputed) data.

|  | **TOTAL** | **N=230** |  |  | **WAI +** | **N=164** |  |  | **WAI -** | **N=63** |  |  |
| --- | --- | --- | --- | --- | --- | --- | --- | --- | --- | --- | --- | --- |
|  | **N** | **%** | **Mean** | **SD** | **N** | **%** | **Mean** | **SD** | **N** | **%** | **Mean** | **SD** |
| **Work Ability Index (7-49)** | 227 | 98.7% | 39.2 | 5.4 | 164 | 100.0% | 41.8 | 3.1 | 63 | 100.0% | 32.3 | 3.7 |
| Poor (7-27) | 7 | 3.0% |  |  |  |  |  |  |  |  |  |  |
| Moderate (28-36) | 56 | 24.3% |  |  |  |  |  |  |  |  |  |  |
| Good (37-43) | 115 | 50.0% |  |  |  |  |  |  |  |  |  |  |
| Excellent (44-49) | 49 | 21.3% |  |  |  |  |  |  |  |  |  |  |
| **Personal characteristics** |  |  |  |  |  |  |  |  |  |  |  |  |
| Gender (% male) | 230 | 89.6% |  |  | 164 | 89.6% |  |  | 63 | 90.5% |  |  |
| Age (yr) | 230 |  | 52.9 | 6.7 | 164 |  | 52.2 | 6.7 | 63 |  | 54.5 | 6.4 |
| Affiliation duration (yr) | 230 |  | 22.5 | 10.7 | 164 |  | 22.2 | 10.5 | 63 |  | 23.9 | 10.5 |
| Contract hours / 4 weeks (hr) | 230 |  | 141.6 | 15.1 | 164 |  | 141.6 | 15.2 | 63 |  | 141.6 | 15.4 |
| Educational level, low | 230 | 74.3% |  |  | 164 | 74.4% |  |  | 63 | 76.2% |  |  |
| **Biometric data** |  |  |  |  |  |  |  |  |  |  |  |  |
| Cholesterol (mmol/l) | 218 |  | 5.3 | 0.9 | 154 |  | 5.3 | 0.9 | 61 |  | 5.4 | 0.9 |
| Glucose (mmol/l) | 221 |  | 5.9 | 1.7 | 157 |  | 5.8 | 1.8 | 61 |  | 6.1 | 1.5 |
| Systolic BP (mm Hg) | 221 |  | 140.8 | 17.8 | 157 |  | 142.1 | 18.7 | 61 |  | 137.3 | 15.3 |
| Diastolic BP (mm Hg) | 221 |  | 82.7 | 9.8 | 157 |  | 82.6 | 10.2 | 61 |  | 82.7 | 8.6 |
| Resting heart rate (bpm) | 220 |  | 71.5 | 12.0 | 157 |  | 71.4 | 11.6 | 60 |  | 71.4 | 13.0 |
| Body length (m) | 222 |  | 175.6 | 8.7 | 158 |  | 176.0 | 8.5 | 61 |  | 174.7 | 9.0 |
| Body weight (kg) | 222 |  | 85.9 | 15.6 | 158 |  | 85.5 | 15.8 | 61 |  | 87.6 | 15.3 |
| Fat percentage (%) | 222 |  | 27.4 | 7.1 | 158 |  | 27.0 | 7.3 | 61 |  | 28.6 | 6.3 |
| **Health** |  |  |  |  |  |  |  |  |  |  |  |  |
| Smoking, yes | 198 | 35.2% |  |  | 144 | 36.6% |  |  | 51 | 31.7% |  |  |
| Alcohol use, yes | 230 | 77.4% |  |  | 164 | 79.9% |  |  | 63 | 71.4% |  |  |
| Healthy eating habits, yes | 228 | 47.4% |  |  | 164 | 47.0% |  |  | 63 | 49.2% |  |  |
| **Functional capacity*** |  |  |  |  |  |  |  |  |  |  |  |  |
| Aerobic capacity (ml/min/kg) | 180 |  | 31.3 | 8.5 | 128 |  | 32.0 | 8.9 | 50 |  | 29.5 | 7.0 |
| Lifting low (kg) | 171 |  | 32.1 | 10.1 | 121 |  | 33.5 | 10.2 | 48 |  | 28.4 | 9.1 |
| Lifting high (kg) | 162 |  | 16.9 | 4.7 | 115 |  | 17.3 | 4.4 | 45 |  | 15.9 | 5.5 |
| Carrying (kg) | 166 |  | 36.9 | 9.1 | 117 |  | 37.9 | 9.0 | 47 |  | 35.0 | 8.8 |
| Overhead work (s) | 171 |  | 221.0 | 75.9 | 122 |  | 235.9 | 70.8 | 47 |  | 179.0 | 73.6 |
| Forward bent work (s) | 177 |  | 244.9 | 78.9 | 126 |  | 254.5 | 70.6 | 48 |  | 216.3 | 93.6 |
| Repetitive bending (s) | 171 |  | 47.9 | 8.2 | 119 |  | 47.1 | 7.3 | 49 |  | 49.7 | 10.1 |
| Trunk rotation right (s) | 189 |  | 68.2 | 13.3 | 130 |  | 66.6 | 10.9 | 56 |  | 72.7 | 14.5 |
| Trunk rotation left (s) | 190 |  | 68.3 | 12.2 | 131 |  | 66.4 | 11.2 | 56 |  | 72.5 | 13.5 |
| Hand grip strength (kgf) | 215 |  | 49.0 | 10.9 | 151 |  | 49.3 | 10.8 | 61 |  | 48.3 | 11.2 |
| **VBBA** |  |  |  |  |  |  |  |  |  |  |  |  |
| High physical workload, yes | 230 | 43.5% |  |  | 164 | 39.6% |  |  | 63 | 54.0% |  |  |
| High mental workload, yes | 230 | 17.4% |  |  | 164 | 14.6% |  |  | 63 | 23.8% |  |  |
| Need for recovery (0-5) | 230 |  | 1.2 | 1.4 | 164 |  | 0.9 | 1.2 | 63 |  | 2.1 | 1.7 |
